# Supplementary material for: Validation of simulated training sets using a convolutional neural network for isotope identification in urban environments
Source: PLoS One. 2025 Jun 9;20(6):e0323053. doi: 10.1371/journal.pone.0323053 (PMC12148181; doi:10.1371/journal.pone.0323053)
Supplement: Attachment — Submitted filename: Response to Reviewers.pdf [file pone.0323053.s001.pdf]

UNIVERSITY OF SURREY

Faculty of
Engineering & Physical
Sciences

Department of Physics
Guildford, Surrey GU2 7XH UK

28th February 2025

PLOS One

Dear Editor,

Thank you for forwarding the reviewer comments on our manuscript now titled “Validation of simulated training sets using a convolutional neural network for isotope identification in urban environments”. We sincerely appreciate the time and effort the reviewers have taken to evaluate our work and provide constructive feedback. Their insights have been invaluable in improving the quality and clarity of our work.

We have carefully considered each comment and made the necessary revisions. A detailed response to each point is provided in the table below. Unfortunately, we are unable to release the data used for this project as it is held by AWE Nuclear Security Technologies. We have provided a link to request access for this data in the citations.

We hope that our revisions and explanations adequately address the reviewers’ concerns. Thank you for considering our revised manuscript. We look forward to your response.

Kind Regards,

Dr Luke Lee-Brewin MInstP
University of Surrey, Department of Physics

Dr Caroline Shenton-Taylor CPhys MInstP FHEA
University of Surrey, Department of Physics

UNIVERSITY OF SURREY

Faculty of
Engineering & Physical
Sciences

Department of Physics
Guildford, Surrey GU2 7XH UK

Reviewer 1

The title is not appropriate at all, it should be revised!	The title has been renamed to: Validation of simulated training sets using a convolutional neural network for isotope identification in urban environments
There is no comparison is clear between experimental and simulated results in the Abstract	We have made this clearer by including the lowest class prediction accuracies for the simulated dataset and the experimental dataset.
The abstract is not appropriate at all, it should be revised and rewritten (Background, methodology, results, conclusion)	We have rewritten it to address these concerns.
The methodology for combining simulated and experimental gamma spectra is intriguing but needs clearer explanation. For instance, how were discrepancies between simulated and experimental data handled during training?	The purpose of this work is to generate representative, simulated spectra without requiring new, experimental data. Discrepancies between simulated and experimental data are the focus of this research.
While the SIGMA dataset is mentioned, additional details about its composition, diversity of isotopes, and representativeness of real-world scenarios would strengthen the study.	Full analysis of the SIGMA dataset is a multi-year venture, currently being undertaken by a group of university and industry partners as part of the SIGMA data challenge. We have explained more about the SIGMA data challenge and cited the link to request access to the data. **Lines 21-29**
The study would benefit from benchmarking the CNN against traditional machine learning or other state-of-the-art deep learning models for isotope identification.	The validation of this methodology leads onto the testing of larger scale models trained using this methodology. Benchmarking against other isotope identification techniques will be carried out once a larger scale model has been developed. We have addressed this in the conclusion. **Lines 226-228**
Were any pre-processing techniques applied to the gamma spectra before feeding them into the CNN?	The gamma spectra were normalised before being using to train the model. We have emphasised this and explained why no further pre-processing was completed at this time. **Lines 100-105**
I recommend to cite the next article that more appropriate for your work (Measurement 168, 108456, 2021)	Thank you for highlighting this piece of work, we will benefit from this in our forthcoming research papers.
What steps were taken to ensure that the CNN generalizes well to unseen datasets?	Regularisation was used to avoid overfitting and extensive hypertuning was performed to optimise the structure of the datasets. All testing was performed on experimentally collected data that the model has not been trained on.
The presentation of the work including heading titles should be well revised	We have made the subheadings more concise.

UNIVERSITY OF SURREY

Faculty of
Engineering & Physical
Sciences

Department of Physics
Guildford, Surrey GU2 7XH UK

Reviewer 2

Line 94: "This subset is will contain a higher ratio of alarms to." The grammatical issue should be correct for more clarity.	We have corrected this sentence.

Reviewer 3

On page 8, the authors state "... more than 5 standard deviations above the average number of counts for a given day".<br>What is the method of calculating the average number of counts for a given day? For example, is it a rolling average that continues until the alarm occurs, or is it a total average across the entire day? If it is the latter, how may this method "be effectively deployed to identify isotopes in real time"?	We have explained this in more detail including some discussion on the limitations of this methodology. **Lines 168-171**
Regarding the Geant4 simulation: Are more details available regarding how the simulation was performed? E.g., was the distance between the source and the detector always stationary? Were there any other obstructions other than the aluminum shielding? Were there any possibilities of gamma ray scattering within the environment?	We have elaborated on the need to reduce computational overhead as the number of spectra simulated will scale with the number of isotopes newer models are trained to identify. We have also commented on other work being undertaken about modelling backscatter in GEANT4. **Lines 207-220**
Would it be possible for the authors to comment on how the manual identification-related errors in the k-means clustering procedure could potentially influence the reported accuracies of the identification network?	This is an excellent point. We have commented on the prediction accuracy and made it clear that this might alter the results we have presented by a small amount. **Lines 194-200**
It may be beneficial to provide insights on a more impactful method other than the 5-sigma threshold technique for identifying alarms: For example, an unsupervised autoencoder architecture (such as ARAD: https://doi.org/10.1016/j.engappai.2022.104761, among others). I would recommend providing brief information on alternatives and whether or not they would be applicable in this case.	We have cited this paper and commented on other anomaly detection techniques in the conclusion.<br>**Lines 180-182**
The first sentence of page two states: " In 2018 the Atomic Weapons Establishment (AWE) created using a subset of the SIGMA dataset."<br>I presume this should read "... created a dataset using a subset …"; but, in any case, I believe this sentence should be revised.	We have corrected this sentence.
